# Designing the relational team development intervention to improve management of mental health in primary care using iterative stakeholder engagement

**DOI:** 10.1186/s12875-019-1010-z

**Published:** 2019-09-06

**Authors:** Danielle F. Loeb, Danielle M. Kline, Kurt Kroenke, Cynthia Boyd, Elizabeth A. Bayliss, Evette Ludman, L. Miriam Dickinson, Ingrid A. Binswanger, Samantha P. Monson

**Affiliations:** 1 Division of General Internal Medicine, Department of Medicine, University of Colorado School of Medicine, Academic Office 1; Mailstop B180; 12631 East 17th Ave, Aurora, CO 80045 USA; 2 Indiana University School of Medicine, Indianapolis, IN USA; 3 John Hopkins University School of Medicine, Baltimore, MD USA; 4 Kaiser Permanente Institute for Health Research, Aurora, CO USA; 5 Kaiser Permanente Washington Health Research Institute, Seattle, WA USA; 6 Department of Biostatistics & Informatics, Colorado School of Public Health; Department of Family Medicine, University of Colorado, Aurora, CO USA; 7 Lowry Family Health Center, Denver Health, Denver, CO USA

## Abstract

**Background:**

Team-based models of care are efficacious in improving outcomes for patients with mental and physical illnesses. However, primary care clinics have been slow to adopt these models. We used iterative stakeholder engagement to develop an intervention to improve the implementation of team-based care for this complex population.

**Methods:**

We developed the initial framework for Relational Team Development (RELATED) from a qualitative study of Primary Care Providers’ (PCPs’) experiences treating mental illness and a literature review of practice facilitation and psychology clinical supervision. Subsequently, we surveyed 900 Colorado PCPs to identify factors associated with PCP self-efficacy in management of mental illness and team-based care. We then conducted two focus groups for feedback on RELATED. Lastly, we convened an expert panel to refine the intervention.

**Results:**

We developed RELATED, a two-part intervention delivered by a practice facilitator with a background in clinical psychology. The facilitator observes PCPs during patient visits and provides individualized coaching. Next, the facilitator guides the primary care team through a practice change activity with a focus on relational team dynamics.

**Conclusion:**

The iterative development of RELATED using stakeholder engagement offers a model for the development of interventions tailored to the needs of these stakeholders.

**Trial registration:**

Not applicable.

**Electronic supplementary material:**

The online version of this article (10.1186/s12875-019-1010-z) contains supplementary material, which is available to authorized users.

## Background

Patients with mental illness and chronic physical conditions are at high risk of poor quality of life and have high medical costs, poor outcomes, and high mortality [1–15]. Team-based models of care founded on the chronic care model [16–21], such as the collaborative care model [22–28] and the Patient-Centered Medical Home (PCMH) [29–34], have gained broad acceptance as efficacious population-based primary care models to improve psychiatric and medical outcomes for patients with concomitant mental and physical illnesses. However, primary care clinics have been slow to adopt these evidence-based models [35–37].

Practice facilitation improves implementation of team-based care models but it often does not directly address relational aspects of team culture that can be integral to sustainable practice change. Practice facilitators are health care professionals trained in primary care practice improvement methods who facilitate system-level changes [38–40]. Practice facilitation has been shown to improve multiple aspects of team-based care: 1) improved communication across different specialties [38, 41, 42]; 2) increased adoption of practice change [43, 44]; and 3) consensus building [45]. One large, randomized controlled trial found that practice facilitation increased “adaptive reserve”, defined as “the ability to make and sustain change.” [40] Practice facilitation is also an effective training model for practicing healthcare providers [44, 46]. Practice facilitators can be used to train providers in population-based and algorithmic management of mental illness as they often have expertise in specific areas of primary care practice [46, 47].

Although practice facilitation effectively focuses on the practical aspects of practice change, it often does not focus on the relational aspects of team culture. Team climate and relational coordination predict quality of chronic disease care [48–51]. Relational Coordination is a theory of organizational management that focuses on the interdependent relationships among people working in teams. It identifies three relational domains that affect team functioning and require nuanced communication skills: shared goals, shared knowledge, and mutual respect. Successful implementation and sustainability of team-based models of care require high levels of relational coordination for individuals from differing professional backgrounds to effectively deliver patient care. However, most healthcare professionals, including primary care providers (PCPs), receive only minimal training in team-based communication skills, leaving them ill equipped to foster strong relational coordination within their team.

Although PCPs generally prefer team-based care for patients with mental illness, transitioning to team-based care can be challenging [52–66]. Additionally, PCPs also have high levels of uncertainty in their knowledge and skills in caring for patients with mental illness and need more support in caring for these complex patientspatients [67–71]. In a qualitative study of 15 internal medicine PCPs, participants expressed a high level of distress with respect to practicing beyond the scope of their knowledge and clinical skills. They also described challenges in communicating effectively with their patients and consultants when treating patients with concomitant mental and physical illness [67]. Other studies also found high levels of uncertainty in PCPs’ clinical skills to diagnose and treat mental illness [70, 71]. This uncertainty can deter engagement in the management of mental illness and lead to suboptimal care. Thus, PCPs need additional training in both the diagnosis and treatment of mental illness and in working effectively in a team setting.

In order to create meaningful and lasting improvements to interdisciplinary primary care teams, we developed Relational Team Development (RELATED) from principles of practice facilitation and psychology clinical supervision. It aims to improve the care of patients with comorbid mental and physical illness in the primary care setting by addressing dysfunctional team dynamics and closing gaps in PCP skills and knowledge. In the development process we repeatedly revised the intervention with feedback from representatives from the target population, local stakeholders, and national experts. In this paper, we describe the iterative development process in which we incorporated expert and stakeholder feedback and modified the RELATED intervention accordingly.

## Methods

**Fig. 1:**
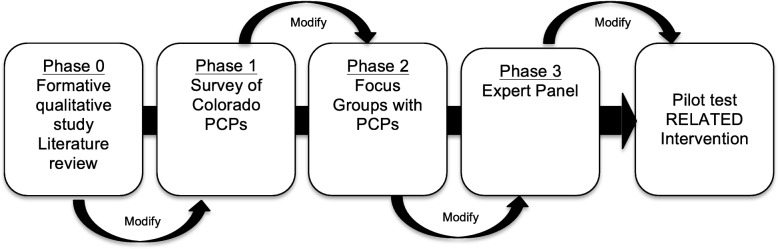
Iterative Development of RELATED Intervention

The development of RELATED began with phase 0, which was generative work through a qualitative research study to identify needs of the target population (i.e. PCPs) and a literature review to identify core components of an intervention. After delineating this initial framework, we developed RELATED in three phases (see Fig. 1). In the first phase, we conducted a survey to identify factors associated with self-efficacy in team-based care and mental illness management, as well as to assess self-efficacy in these constructs. In the second phase, we conducted two focus groups with PCPs in a clinic similar to the planned intervention clinic to gain feedback on the planned intervention components. In the third phase, we convened an expert panel to further refine the pilot intervention. The Colorado Institutional Review Board (COMIRB) at the University of Colorado (Aurora, Colorado) approved all study procedures.

### Phase 0 preparatory work. Qualitative study and literature reviews

The initial framework for RELATED was based on preliminary qualitative research in which we used in-depth semi-structured interviews of 15 internal medicine PCPs working in two academic and three community health clinics to examine perceptions of patient complexity, including co-morbidity of mental and physical illnesses, and to identify domains that PCPs felt affected care of patients they defined as complex [67–69]. We invited 34 academic clinic and 28 community health clinic physicians by email to participate in the interviews. We used non-probabilistic sampling to achieve an even distribution of participants by gender, years in practice, and type of practice [67]. (See Additional file 1: Phase 0 Interview Guide) We learned: 1) PCPs have variable competence levels in treating mental illness; 2) Both clinic-level and larger system barriers inhibit PCPs’ ability to care for patients with mental and physical illness; 3) PCPs need additional training in patient communication; and 4) PCPs prefer didactic and experiential training in the management of patients with mental and physical illness [68]. Informed by the needs expressed by PCPs in their management of patients with mental and physical illness in the primary care setting, we performed a review of practice facilitation and psychology clinical supervision models. We aimed to use aspects of psychology clinical supervision to tailor a practice facilitation model to intervene on knowledge and skill gaps of PCPs in caring for the complex patient population.

### Phase 1 methods. Survey of Colorado PCPs

To identify factors associated with PCP self-efficacy in the management of mental illness in a team-based model of care, we conducted a cross-sectional mail survey of currently practicing family medicine and internal medicine physicians in the state of Colorado (*N* = 900). In the survey, we validated two self-efficacy scales: Team-Based Care (TBC) and Mental Illness Management (MIM). The scales were based on Bandura’s social cognitive theory. The MIM scale addressed the content areas of diagnosis and treatment of depression, generalized anxiety disorder, and bipolar depression, and management of concomitant psychiatric and medical illness. The TBC scale addressed the following content areas based on NCQA PCMH 2011 standard: communication within the team, care coordination, population management, self-management support, and continuity of care [72]. They were scored on a 0 to 10 Likert-type scale where 0 is “not at all confident” and 10 is “extremely confident”. The scales were validated using factor analysis, Cronbach’s alpha, and comparisons to existing measures. Then, using multivariable linear regression, the TBC (7 items) and MIM (10 items) scores were assessed as predictors of communication self-efficacy, attitudes toward team care, team climate, implementation of team care, knowledge and experience treating mental illness, and practice characteristics. (See Additional file 2: Phase 1 Survey Instrument) The participants, data collection, and data analysis of this study have been previously described [73, 74].

### Phase 2 methods. Focus groups with PCPs

After developing the initial framework of the intervention and revising it based on the survey of PCPs, we conducted focus groups with local PCPs from local stakeholders similar to those who would receive the intervention. Focus groups were used to obtain feedback on the modified RELATED intervention that emerged following survey results. We chose focus groups rather than key-informant interviews so that PCPs could interact and build upon each other’s ideas.

#### Participants

We recruited PCPs from the University of Colorado Anschutz Outpatient Pavilion Internal Medicine clinic via email and clinic team announcement. Like the clinic for the planned intervention, all PCPs were associated with the University of Colorado School of Medicine. However, they work in a different hospital system. All PCP’s in the clinic were invited to participate.

#### Data collection

The Principal Investigator (DL) conducted two 60-min focus groups over lunch and provided food as an incentive. DL is a PCP who works in the clinic in which the focus groups were conducted. The focus groups were semi-structured to allow for a systematic and flexible approach [15]. The interview guide was created by DL. Domains addressed in the interview guide included: experiences of team-based care in current clinic, opinions of feasibility and acceptability of components of RELATED intervention, expected barriers and facilitators to implementation of the intervention, and suggested changes to intervention components. (See Additional file 3: Phase 2 Interview Guide) Participants were encouraged to express themselves in their own terms [16]. The focus groups were audio recorded, transcribed and de-identified.

#### Data analysis

Two research team members (DL and DK) reviewed the transcripts to identify thematic elements in a content analysis. They then outlined the most prominent themes. These results were summarized for the full research team.

### Phase 3 methods. Expert panel

#### Participants

National experts were recruited to guide further refinement of the RELATED intervention and pilot study. Members of the expert panel were chosen based on expertise in the subject area and methods planned for the RELATED intervention. They were chosen to complement the expertise of the research team. The research team had expertise in patients with multiple chronic conditions (LB and DL), mental health in primary care (EL and FD) the implementation of practice change (DN), study design (IB) and biostats methods (MD and LC). Three experts brought specialized expertise in the Chronic Care Model (MP); designing interventions for patients with multiple chronic conditions (CB); and practice measures for mental illness in primary care (KK). All experts who were invited agreed to participate in the panel. They were also deliberately selected to represent both the mental health and medical professions, mirroring the RELATED target patient populations. Experts were recruited through emails and follow-up phone conversations. The experts joined the research team, which beginning with development and continuing through the completion of pilot testing.

#### Data collection

We conducted two 90-min calls with the full research team, including the expert panel. In advance of the first meeting, all participants received a description of the RELATED intervention in its most updated format, as well as results from the qualitative study, survey, and focus group. The experts were asked to provide feedback from their area of expertise during the first meeting. After this discussion, the PI worked with the research team to modify the RELATED intervention. This modified version was sent to the full research team prior to the second call. The group discussed the modifications and the experts made final recommendations. The PI led both discussions, which were audio recorded, transcribed, and de-identified.

#### Data analysis

Two research team members (DL and DK) reviewed the transcripts of the expert panel calls and outlined key findings. These were consolidated into themes of recommended changes to the RELATED intervention.

## Results

### Phase 0 pre-work. Qualitative study and literature reviews

**Fig. 2:**
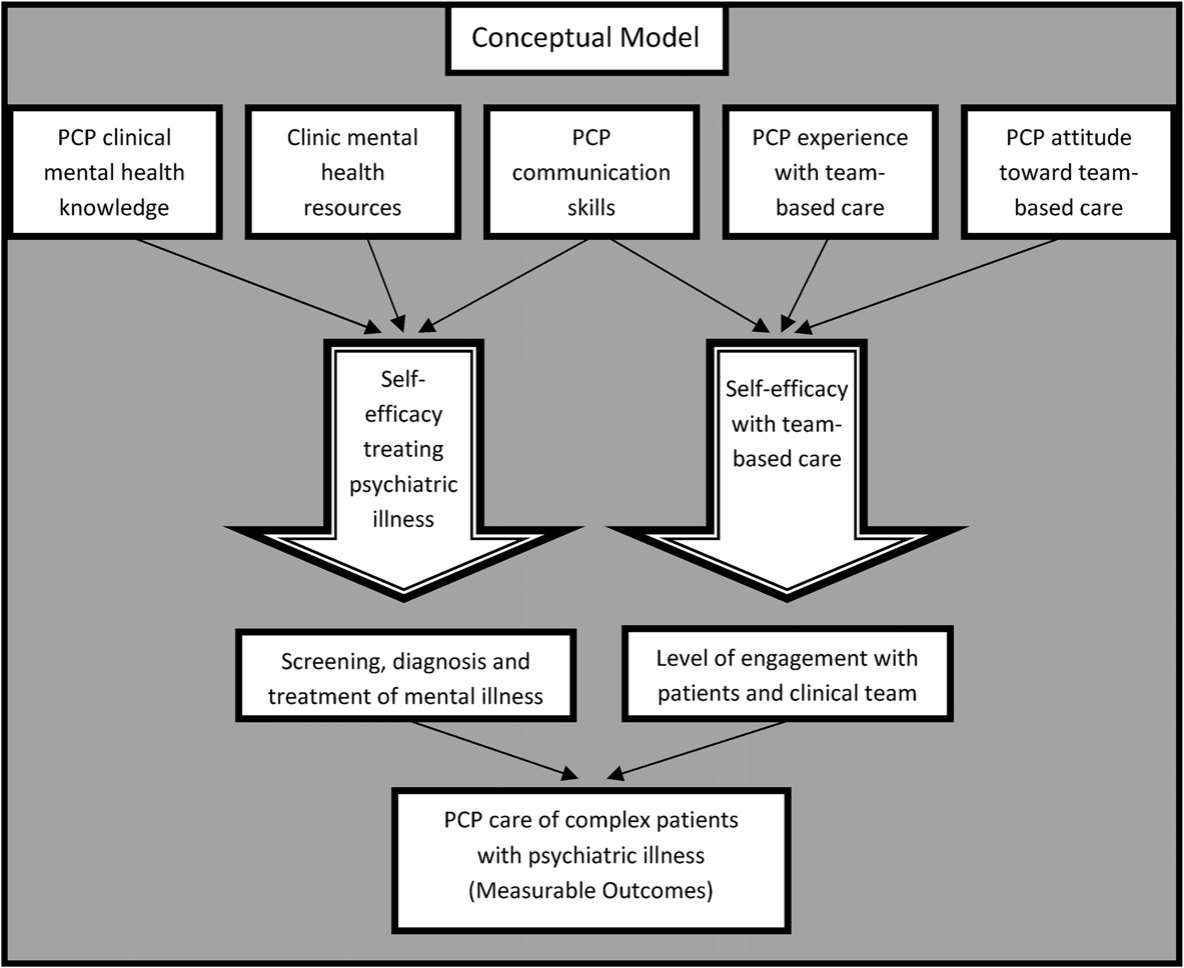
Conceptual Model of Role of PCP Self-efficacy in the Care of Complex Patients

Results from our qualitative study of PCPs enabled development of a conceptual model for the role of PCP self-efficacy in managing mental illness in a team-based setting (Fig. 2). PCPs in the study expressed poor self-efficacy in managing patients with mental illness in their primary care practices. Therefore, we focused on self-efficacy in the conceptual model. Self-efficacy is a measurable, modifiable trait associated with provider behavior. Provider self-efficacy in performing preventive health care education has been linked to performance of guideline-concordant care [75, 76]. We hypothesized that PCP clinical knowledge, mental health resources available in clinic and PCP communication skills influenced PCP MIM self-efficacy and that PCP communication skills, PCP experience with team-based care, and PCP attitudes toward team-based care influenced PCP TBC self-efficacy. These factors became the preliminary targets of the RELATED intervention, to be tested subsequently through the survey of Colorado PCPs (see below).

**Fig. 3:**
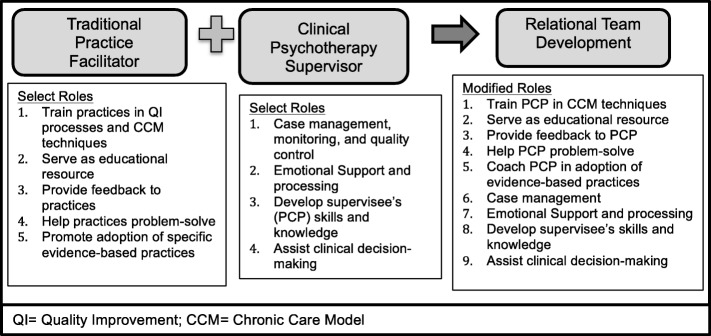
Conceptualization of Role of Practice Facilitator in RELATED

We then conducted literature reviews of practice facilitation and clinical psychology supervision*.* These constructs were selected due to their known impact on factors similar to those hypothesized in our PCP self-efficacy model. Practice facilitators are health care professionals trained in primary care practice improvement methods who work with clinics to facilitate system-level changes [77]. They have been shown to increase the adoption of evidence-based care in primary care practices [38, 40, 77]. Psychology clinical supervision leads to increased knowledge of and increased acquisition of psychotherapy skills among both psychotherapists and psychiatric nurses [78, 79]. Figure 3, depicts how practice facilitation and psychology clinical supervision interventions were combined to form the basis of the RELATED intervention.

#### Initial RELATED intervention

We used the results of the qualitative study to define targets of the intervention: PCP clinical knowledge, mental health resources available in clinic, PCP communication skills, PCP experience with team-based care, and PCP attitudes toward team-based. We then identified and combined core components of practice facilitation and psychology clinical supervision in the initial RELATED intervention. Figure 3 illustrates the resulting conceptualization of the practice facilitator incorporating roles of traditional practice facilitators and clinical psychotherapy supervision roles. The practice facilitator would have a clinical background in psychology and training in traditional practice facilitation. In the intervention, this practice facilitator would observe PCPs in visits with patients with co-morbid mental and physical illnesses and provide tailored feedback on the above factors.

### Phase 1 results. Survey of Colorado PCPs

We had a 49% response rate (441 of 900 surveyed) from PCPs in diverse practice settings. On a 0 to 10 Likert scale mean scores (standard deviation) were 7.7 (1.7) and 7.1 (1.4) for the TBC and MIM scales, respectively. Cronbach’s alpha for the TBC scale was 0.94 and MIM was 0.88 [73]. In multivariable analysis, greater TBC self-efficacy was significantly associated with more favorable attitudes toward team-based care, the presence of a health psychologist in clinic, and greater communication self-efficacy. Solo practice was associated with lower TBC self-efficacy. Greater MIM was significantly associated with higher score on mental illness management knowledge assessment, greater communication self-efficacy and access to psychology referral. Feeling “not at all” or “slightly” prepared to manage bipolar disorder at residency completion was associated with lower MIM self-efficacy [74]. Finally, the multivariable analysis supported a focus on PCP mental illness management knowledge, communication skills, attitudes toward team-based care, and experience of treating serious mental illness such as bipolar disorder.

#### Modified RELATED intervention

These survey results supported the conceptual model of PCP self-efficacy developed in the qualitative study. We used these results to design content for the practice facilitator to emphasize during the one-on-one coaching sessions. We identified areas of focus for the intervention as communication skills, MIM assessment and management, and attitudes toward engaging their interdisciplinary team.

### Phase 2 results. Focus groups with PCPs

We completed two focus groups with a total of 9 PCPs and 1 care manager. Participants were 80% female, 80% Caucasian, 90% non-Hispanic, and averaged an age of 44 (+ 6.7). Of the 9 PCPs, 78% completed residency between 10 and 19 years ago, the other 22% of PCPs completed residency between 5 and 9 years ago. Overall, PCPs had a positive overall impression of the proposed intervention. Participants thought the one-on-one coaching sessions could reasonably be expected to impact PCP behavior with their patients, but they were unlikely to have meaningful effect on PCP interactions with their team. They felt it would help them manage patients with mental illness. They also thought it would be therapeutic for the stress of managing patients, calling it “therapy for doctors”. However, they did not think it would help them work within a team and participate in practice improvement. They felt that a practical team activity would be necessary to influence their ability to work with their teams.

#### Modified RELATED intervention

From the feedback in the focus groups the idea collectively emerged to add a practice change activity to RELATED in which PCPs could engage with their interdisciplinary team members. This larger clinic-wide activity would provide a real-time opportunity for PCPs to work within their teams to identify a gap in the clinic’s care of patients with mental and physical illness. The team would work together on developing and implementing a clinical practice improvement to address the identified gap in care. This activity would offer the practice facilitator the opportunity to observe and intervene on team dynamics in real time.

### Phase 3 results. Expert panel

The RELATED intervention was finalized and made pilot-ready through the expert panel. We developed a detailed structure for the one-on-one coaching sessions and the practice change activity. Burden for the PCPs and clinical team was a primary concern of the expert panel. The group decided that for one-on-one coaching sessions for each PCP should lead the intervention. In addition to the direct intervention effects, these sessions would provide the practice facilitator with the opportunity to learn about clinic culture and develop rapport with the PCPs. The group decided six meetings would be sufficient for the intended impact without creating undue burden on the practice. They also recommended expansion of didactic material out of the one-on-one coaching sessions to be integrated into the Practice Change Activity Team (PCAT) with the whole team, providing important educational content and a novel collective learning experience. Finally, they noted the absence of patients in the intervention and identified the PCAT as an opportunity for PCPs and staff to benefit from their important feedback on the selected change.

#### Modified RELATED intervention

The one-on-one coaching sessions were truncated to create time for the practice change activity. The latter was termed PCAT and fully developed as a lab for working on team dynamics, including hierarchical power, and applied quality improvement learning. Detailed results of the expert panel can be found in Table 1, which details the components of the RELATED intervention.

**Table 1:** Components of Relational Team Development (RELATED)

	PCP Clinical Supervision and Coaching (Coaching)	Practice Change Activity Team (PCAT)
Description	Practice facilitator shadows PCPs in 4+ visits with patients with co-morbid mental and physical illnesses - Use clinical psychology and coaching techniques in one-on-one debriefs with PCPs after visits	Practice facilitator guides clinical team (with PCP participants) through a practice change activity focused on the care of patients with co-morbid mental and physical illnesses -In this process maladaptive team dynamics are identified and addressed
Content	-Mental health diagnosis and treatment -Patient and team communication skills -Tailored to individual goals -Personal transformation focus	-Quality improvement methods -Evidence-based practices for team-based care -Team dynamics
Mechanism of Action	-Therapeutic relationship -Personal growth -Skill building	Quality improvement process as a lab for applied team dynamic learning
Participants	-PCPs -Patients whose visits are observed	-PCPs -Staff representatives -Leadership -Patient representatives (coaching component)
Timeline	4 or more meetings over weeks 1–8 of RELATED	6–8 meetings (2 optional didactics) over weeks 9–16 of RELATED

## Discussion

We describe the iterative development of RELATED as an intervention to enhance the care of patients with mental illness in the primary care setting through one-on-one PCP coaching and a team-based practice change activity. A practice facilitator with a background in clinical psychology delivers the intervention, which combines core components of psychology clinical supervision and practice facilitation into the aforementioned framework. The initial RELATED intervention, which included only the one-on-one coaching of PCPs, was substantively different than the ultimate pilot-ready version, which also included patients and interdisciplinary staff in a practice change activity. This significant change exemplifies the powerful effect of stakeholder involvement.

The importance provider of engagement is increasingly recognized as fundamental to developing research that will be most useful in clinical practice [80, 81]. Shelef, et.al. have described a similar method of engaging stakeholders to meet the needs of a target population [82]. They present a model of collaboratively refining the intervention with repeated input from stakeholders at multiple levels, including representatives of the target population, local stakeholders, and national advisors to develop an asthma intervention [82]. Our development of RELATED can be seen as an example of utilizing this method of engaging multilevel stakeholders. As the primary subjects of the RELATED intervention, we saw PCPs as a key target population in the development of the intervention. We incorporated PCP experience and perspectives from the in the formative development of the framework through qualitative interviews and in the larger survey. We sought local PCP stakeholder feedback through focus groups in a clinic similar to that of the planned pilot intervention. We, then, incorporated feedback from national advisors though our expert panel. This process may be useful to researchers seeking to design interventions with high acceptability in clinical settings.

Although we employed a multilevel stakeholder process involving an expert panel, we did not utilize the Delphi Technique for that panel. The Delphi Technique was initially developed by the RAND Corporation as a method of iteratively sampling expert opinion to eventually come to consensus. One of the primary benefits of the Delphi Techniques is that it allows participants to remain anonymous and, hence, maintains equal representation among experts. [83, 84] We chose to use a more open discussion among experts to encourage common understanding and cross-fertilization of ideas. The rich exchange of ideas in our expert panel led to both a deep understanding of the intention of the RELATED intervention and a valuable and collaborative discussion of suggested changes to meet that goal.

### Limitations

There are several limitations of the iterative stakeholder engagement strategy used to develop the RELATED intervention. In our effort to elicit feedback from local stakeholders, we conducted focus groups with a small number of providers from one urban academic clinic. Alternatively, sampling from non-academic and non-urban settings may have increased the generalizability of the results (i.e., the RELATED intervention). Additionally, non-PCP stakeholders were omitted from the process due to our initial definition of PCPs as the primary target population. The healthcare team, more generally, became the focus of the intervention with the development of the PCAT. Therefore, additional focus groups with interdisciplinary team members could have been useful once the PCAT was added as an intervention component. This additional local stakeholder would have provided opportunity for input on the contents and structure of RELATED from a non-PCP perspective. Further, patient stakeholders were not included in the development of RELATED. Although RELATED does not directly intervene with patients, it does ultimately affect patient care.

### Future directions

Following completion of RELATED development, a partnership was formed with Denver Health, a safety net hospital with a network of neighboring safety net primary care practices, who agreed to host the piloting of RELATED. This partnership provided an immediate opportunity to increase the generalizability of RELATED by testing and refining it for a safety-net hospital. We are pursuing additional partnerships with another network of safety net primary care practices that have rural reach, which would further generalize RELATED’s scope. Finally, funding is being sought to examine the efficacy of RELATED efficacy in bringing about desired change in PCPs and the relational dynamics of the team in which they practice.

## Conclusions

In this manuscript we describe the use of stakeholder engagement in designing RELATED, a brief clinic-wide intervention that combines team-level practice facilitation and PCP-level clinical coaching to help primary care practices implement evidence-based practices for patients with mental illness. In an approach similar to that described by Shelef et.al [82], .we iteratively refined the intervention with repeated input from stakeholders at multiple levels, including representatives of the target population, local stakeholders, and national advisors. This method may serve as a model for the development of interventions to have high acceptability in the primary care setting.

## Additional files

Additional file 1: Phase 0 Interview Guide. (DOC 38kb)

Additional file 2: Phase 1 Survey Instrument. (DOCx 115kb)

Additional file 3: Phase 2 Interview Guide. (DOC 39kb)

## Data Availability

The datasets used and analyzed during the current study are available from the corresponding author on reasonable request.

## Abbreviations

COMIRB: Colorado Institutional Review Board
MIM: Mental Illness Management
PCAT: Practice Change Activity Team
PCMH: Patient-Centered Medical Home
PCP: Primary Care Providers
PI: Principal Investigator
RELATED: Relational Team Development
TBC: Team-Based Care

## References

[1] Rochon PA, Katz JN, Morrow LA (1996). Comorbid illness is associated with survival and length of hospital stay in patients with chronic disability. A prospective comparison of three comorbidity indices. Med Care.

[2] Librero J, Peiro S, Ordinana R (1999). Chronic comorbidity and outcomes of hospital care: length of stay, mortality, and readmission at 30 and 365 days. J Clin Epidemiol.

[3] Fortin M, Bravo G, Hudon C (2006). Relationship between multimorbidity and health-related quality of life of patients in primary care. Qual Life Res.

[4] Michelson H, Bolund C, Brandberg Y (2000). Multiple chronic health problems are negatively associated with health related quality of life (HRQoL) irrespective of age. Qual Life Res.

[5] Wolff JL, Starfield B, Anderson G (2002). Prevalence, expenditures, and complications of multiple chronic conditions in the elderly. Arch Intern Med.

[6] Glynn LG, Valderas JM, Healy P (2011). The prevalence of multimorbidity in primary care and its effect on health care utilization and cost. Fam Pract.

[7] Starfield B, Lemke KW, Bernhardt T, Foldes SS, Forrest CB, Weiner JP (2003). Comorbidity: implications for the importance of primary care in ‘case’ management. Ann Fam Med.

[8] Egede LE, Nietert PJ, Zheng D (2005). Depression and all-cause and coronary heart disease mortality among adults with and without diabetes. Diabetes Care.

[9] Katon W, Fan MY, Unutzer J, Taylor J, Pincus H, Schoenbaum M (2008). Depression and diabetes: a potentially lethal combination. J Gen Intern Med.

[10] Connerney Ingrid, Sloan Richard P., Shapiro Peter A., Bagiella Emilia, Seckman Charlotte (2010). Depression Is Associated With Increased Mortality 10 Years After Coronary Artery Bypass Surgery. Psychosomatic Medicine.

[11] Hamer M, Batty GD, Stamatakis E, Kivimaki M. The combined influence of hypertension and common mental disorder on all-cause and cardiovascular disease mortality. J Hypertens. 2010;28(12):2401-6. 10.1097/HJH.0b013e32833e9d7c 20724937

[12] Barth J, Schumacher M, Herrmann-Lingen C (2004). Depression as a risk factor for mortality in patients with coronary heart disease: a meta-analysis. Psychosom Med.

[13] Kreyenbuhl J, Dickerson FB, Medoff DR (2006). Extent and management of cardiovascular risk factors in patients with type 2 diabetes and serious mental illness. J Nerv Ment Dis.

[14] Slomka JM, Piette JD, Post EP (2012). Mood disorder symptoms and elevated cardiovascular disease risk in patients with bipolar disorder. J Affect Disord.

[15] Perron BE, Howard MO, Nienhuis JK, Bauer MS, Woodward AT, Kilbourne AM (2009). Prevalence and burden of general medical conditions among adults with bipolar I disorder: results from the National Epidemiologic Survey on alcohol and related conditions. J Clin Psychiatry.

[16] Wagner EH, Austin BT, Von Korff M (1996). Improving outcomes in chronic illness. Manag care Q.

[17] Wagner EH, Austin BT, Von Korff M (1996). Organizing care for patients with chronic illness. Milbank Q.

[18] Bodenheimer T (2003). Interventions to improve chronic illness care: evaluating their effectiveness. Dis Manag.

[19] Bodenheimer T (2006). Primary care--will it survive?. N Engl J Med.

[20] Bodenheimer T, Pham HH (2010). Primary care: current problems and proposed solutions. Health Aff (Millwood).

[21] Bodenheimer T, Wagner EH, Grumbach K (2002). Improving primary care for patients with chronic illness: the chronic care model, part 2. JAMA.

[22] Katon WJ, Lin EH, Von Korff M (2010). Collaborative care for patients with depression and chronic illnesses. N Engl J Med.

[23] Institute of Medicine. Improving the Quality of Health Care for Mental and Substance-Use Conditions (2006). 2010/07/30 ed.

[24] Keith RE, Hopp FP, Subramanian U, Wiitala W, Lowery JC (2010). Fidelity of implementation: development and testing of a measure. Implement Sci IS.

[25] Katon WJ, Von Korff M, Lin EH (2004). The pathways study: a randomized trial of collaborative care in patients with diabetes and depression. Arch Gen Psychiatry.

[26] Butler M, Kane RL, McAlpine D, et al Integration of mental health/substance abuse and primary care. Evid Rep Technol Assess *(Full Rep).* 2008(173):1–362. PMC4781124 19408966

[27] Bauer MS, McBride L, Williford WO (2006). Collaborative care for bipolar disorder: part II. Impact on clinical outcome, function, and costs. Psychiatr Serv.

[28] Kilbourne AM, Biswas K, Pirraglia PA, Sajatovic M, Williford WO, Bauer MS (2009). Is the collaborative chronic care model effective for patients with bipolar disorder and co-occurring conditions?. J Affect Disord.

[29] Reid RJ, Coleman K, Johnson EA (2010). The group health medical home at year two: cost savings, higher patient satisfaction, and less burnout for providers. Health Aff.

[30] Iglehart JK (2008). No place like home--testing a new model of care delivery. N Engl J Med.

[31] Grumbach K, Bodenheimer T (2002). A primary care home for Americans: putting the house in order. JAMA J of the Am Med Assoc.

[32] Joint principles of the Patient-Centered Medical Home. Del Med J. 2008;80(1):21–2. 18284087

[33] The patient-centered medical home model. Ann Emerg Med. 2009;53(2):289–91. 10.1016/j.annemergmed.2008.10.020 19167629

[34] Stange KC, Nutting PA, Miller WL (2010). Defining and measuring the patient-centered medical home. J Gen Intern Med.

[35] Audet AM, Davis K, Schoenbaum SC (2006). Adoption of patient-centered care practices by physicians: results from a national survey. Arch Intern Med.

[36] Rittenhouse DR, Casalino LP, Shortell SM (2011). Small and medium-size physician practices use few patient-centered medical home processes. Health Aff.

[37] Rittenhouse DR, Casalino LP, Gillies RR, Shortell SM, Lau B (2008). Measuring the medical home infrastructure in large medical groups. Health Aff.

[38] Nagykaldi Z, Mold JW, Aspy CB (2005). Practice facilitators: a review of the literature. Fam Med.

[39] Van der Wees PJ, Friedberg MW, Guzman EA, Ayanian JZ, Rodriguez HP (2014). Comparing the implementation of team approaches for improving diabetes care in community health centers. BMC Health Serv Res.

[40] Nutting PA, Crabtree BF, Stewart EE (2010). Effect of facilitation on practice outcomes in the National Demonstration Project model of the patient-centered medical home. Ann Fam Med.

[41] Mold JW, Peterson KA (2005). Primary care practice-based research networks: working at the interface between research and quality improvement. Ann Fam Med.

[42] Astrop P (1988). Facilitator--the birth of a new profession. Health visitor.

[43] Hogg W, Baskerville N, Nykiforuk C, Mallen D (2002). Improved preventive care in family practices with outreach facilitation: understanding success and failure. J Health Serv Res policy.

[44] Geboers H, van der Horst M, Mokkink H (1999). Setting up improvement projects in small scale primary care practices: feasibility of a model for continuous quality improvement. Qual Health Care.

[45] Baskerville NB, Hogg W, Lemelin J (2001). Process evaluation of a tailored multifaceted approach to changing family physician practice patterns improving preventive care. J Fam Prac.

[46] Bashir K, Blizard B, Bosanquet A, Bosanquet N, Mann A, Jenkins R (2000). The evaluation of a mental health facilitator in general practice: effects on recognition, management, and outcome of mental illness. Br J Gen Pract.

[47] Knox L, Taylor E, Geonnotti K (2011). Developing and running a primary care practice facilitation program: a how-TO guide (prepared by Mathematica policy research under contract no. HHSA290200900019I TO 5.) AHRQ publication no. 12–0011.

[48] Cramm JM, Nieboer AP (2012). In the Netherlands, rich interaction among professionals conducting disease management led to better chronic care. Health Aff (Millwood).

[49] Bower P, Campbell S, Bojke C, Sibbald B (2003). Team structure, team climate and the quality of care in primary care: an observational study. Qual Saf Health Care.

[50] Deri Armstrong C, Taljaard M, Hogg W, Mark AE, Liddy C (2016). Practice facilitation for improving cardiovascular care: secondary evaluation of a stepped wedge cluster randomized controlled trial using population-based administrative data. Trials.

[51] Liddy C, Hogg W, Singh J (2015). A real-world stepped wedge cluster randomized trial of practice facilitation to improve cardiovascular care. Implement Sci.

[52] Farrar S, Kates N, Crustolo AM, Nikolaou L (2001). Integrated model for mental health care. Are health care providers satisfied with it?. Can Fam Physician Medecin de famille canadien.

[53] Younes N, Passerieux C, Hardy-Bayle MC, Falissard B, Gasquet I (2008). Long term GP opinions and involvement after a consultation-liaison intervention for mental health problems. BMC Fam Pract.

[54] Kilbourne Amy M., Greenwald Devra E., Bauer Mark S., Charns Martin P., Yano Elizabeth M. (2011). Mental Health Provider Perspectives Regarding Integrated Medical Care for Patients with Serious Mental Illness. Administration and Policy in Mental Health and Mental Health Services Research.

[55] Kisely S, Duerden D, Shaddick S, Jayabarathan A (2006). Collaboration between primary care and psychiatric services: does it help family physicians?. Can fam physician Med de Fam canadien.

[56] Gallo JJ, Zubritsky C, Maxwell J (2004). Primary care clinicians evaluate integrated and referral models of behavioral health care for older adults: results from a multisite effectiveness trial (PRISM-e). Ann Fam Med.

[57] Franx G, Oud M, de Lange J, Wensing M, Grol R (2012). Implementing a stepped-care approach in primary care: results of a qualitative study. Implementa Sci IS.

[58] Katon W, Von Korff M, Lin E, Simon G (2001). Rethinking practitioner roles in chronic illness: the specialist, primary care physician, and the practice nurse. Gen Hosp Psychiatry.

[59] St Peter RF, Reed MC, Kemper P, Blumenthal D (1999). The scope of care expected of primary care physicians: is it greater than it should be?. Issue brief.

[60] St Peter RF, Reed MC, Kemper P, Blumenthal D (1999). Changes in the scope of care provided by primary care physicians. N Engl J Med.

[61] Wagner EH (2000). The role of patient care teams in chronic disease management. BMJ.

[62] Wright B, Lockyer J, Fidler H, Hofmeister M (2007). Roles and responsibilities of family physicians on geriatric health care teams: health care team members’ perspectives. Can Fam Physician Med de famille canadien.

[63] Leipzig RM, Hyer K, Ek K (2002). Attitudes toward working on interdisciplinary healthcare teams: a comparison by discipline. J Am Geriatr Soc.

[64] Sommers LS, Marton KI, Barbaccia JC, Randolph J (2000). Physician, nurse, and social worker collaboration in primary care for chronically ill seniors. Arch Intern Med.

[65] Steiner JL, Ponce AN, Styron T, Aklin EE, Wexler BE (2008). Teaching an interdisciplinary approach to the treatment of chronic mental illness: challenges and rewards. Acad Psychiatry J Am Assoc of Direct Psychiatric Res Training and the Assoc for Acad Psychiatry.

[66] Coleman K, Reid RJ, Johnson E (2010). Implications of reassigning patients for the medical home: a case study. Ann Fam Med.

[67] Loeb DF, Bayliss EA, Binswanger IA, Candrian C, deGruy FV (2012). Primary care physician perceptions on caring for complex patients with medical and mental illness. J Gen Intern Med.

[68] Loeb DF, Bayliss EA, Candrian C, deGruy FV, Binswanger IA (2016). Primary care providers’ experiences caring for complex patients in primary care: a qualitative study. BMC Fam Pract.

[69] Loeb D. F., Binswanger I. A., Candrian C., Bayliss E. A. (2015). Primary Care Physician Insights Into a Typology of the Complex Patient in Primary Care. The Annals of Family Medicine.

[70] Oud MJ, Schuling J, Slooff CJ, Meyboom-de JB (2007). How do general practitioners experience providing care for their psychotic patients?. BMC Fam Pract.

[71] Ballester DA, Filippon AP, Braga C, Andreoli SB (2005). The general practitioner and mental health problems: challenges and strategies for medical education. Sao Paulo Med J Rev Paul de Med.

[72] Patient-Centered Medical Home (PCMH) 2011. National Committee for Quality Assurance. http://www.ncqa.org/portals/0/programs/recognition/pcmh/pcmh_2011_faqs_9_24_13.pdf. Published 2016. Accessed.

[73] Loeb DF, Crane LA, Leister E (2017). Development and initial validation of primary care provider mental illness management and team-based care self-efficacy scales. Gen Hosp Psychiatry.

[74] Loeb DF, Leister E, Ludman E (2018). Factors associated with physician self-efficacy in mental illness management and team-based care. Gen Hosp Psychiatry.

[75] Cabana MD, Rand CS, Powe NR (1999). Why don't physicians follow clinical practice guidelines? A framework for improvement. JAMA J of the Am Med Assoc.

[76] Ozer EM, Adams SH, Gardner LR, Mailloux DE, Wibbelsman CJ, Irwin CE (2004). Provider self-efficacy and the screening of adolescents for risky health behaviors. J Adolesc Health Off publication of the Soc for Adolesc Med.

[77] Baskerville NB, Liddy C, Hogg W (2012). Systematic review and meta-analysis of practice facilitation within primary care settings. Ann Fam Med.

[78] Holloway EL, Neufeldt SA (1995). Supervision: its contributions to treatment efficacy. J Consult Clin Psychol.

[79] Bradshaw T, Butterworth A, Mairs H (2007). Does structured clinical supervision during psychosocial intervention education enhance outcome for mental health nurses and the service users they work with?. J Psychiatr Ment Health Nurs.

[80] Hong Yoon Duk, Goto Daisuke, Mullins C Daniel (2017). Querying stakeholders to inform comparative effectiveness research. Journal of Comparative Effectiveness Research.

[81] Concannon TW, Meissner P, Grunbaum JA (2012). A new taxonomy for stakeholder engagement in patient-centered outcomes research. J Gen Intern Med.

[82] Shelef DQ, Rand C, Streisand R (2016). Using stakeholder engagement to develop a patient-centered pediatric asthma intervention. J Allergy Clin Immunol.

[83] Keeney S, Hasson F, McKenna HP (2001). A critical review of the Delphi technique as a research methodology for nursing. Int J Nurs Stud.

[84] Hasson F, Keeney S, McKenna H (2000). Research guidelines for the Delphi survey technique. J Adv Nurs.

