# Supplementary material for: Designing the relational team development intervention to improve management of mental health in primary care using iterative stakeholder engagement
Source: BMC Fam Pract. 2019 Sep 6;20:124. doi: 10.1186/s12875-019-1010-z (PMC6728939; doi:10.1186/s12875-019-1010-z)
Supplement: Supplementary file 1 — Phase 0 Interview Guide. (DOC 38kb) [file 12875_2019_1010_MOESM1_ESM.doc]

*Interview Guide*

**Project Title:** What is a Complex Patient in Primary Care? A Qualitative Study on Physician Perceptions of Complexity and the Role of Mental Illness

**Principal Investigator:** Danielle Loeb, MD

**Working definition:** **A complex patient is defined as a person with two or more chronic conditions where each condition may influence the care of the other condition. This patient may have other factors such as age, race, gender and psychosocial issues that also influence the morbidity associated with their chronic conditions.**

In this study we are specifically exploring the role mental health conditions may play in the care of complex patients.

This study explores a fairly new area of research. We want to begin to understand the perceptions of primary care physicians regarding complex patients. Clearly, there are no right or wrong answers—we are just interested in your experience.

***INTRODUCTION:*** *I’m going to ask you some questions about your experiences in dealing with complex patients. Our hope is to learn more about your experiences with complex patients. We expect this discussion to take between 45 minutes and an hour.*

*Before we start, I need to make sure you are aware of the following:*

- *Your participation is voluntary, you do not have to answer any question that makes you uncomfortable, and you may stop at anytime.*
- *Your responses will be recorded in a manner that will maintain your anonymity and confidentiality.*
- *If you have any concerns about this study, you may contact the Primary Investigator –Danielle Loeb (if not the interviewer) or COMIRB (*303-724-0155).

*[Obtain verbal consent] Do you consent to go ahead with the interview?*

*[If no] Thank you for your time.*

*[If yes] Do you have any questions before we begin?*

***BEGIN RECORDING***

**Opening Questions:**

1. Broadly speaking, what do you think about this definition? (ok to repeat for them, give them a copy)
2. ---(probe) If people have trouble giving a definition—ask/explore why it is so hard explore. Ask them to tell you what they are struggling with
3. ---(probe) If still difficulty talking about complexity-- Tell me a story about a recent encounter with a patient you don’t think of as “complex”. Then probe to explore what patient factors make this person not complex.
4. I would like to explore this definition in a little bit more detail: I would like to explore what factors most impact the care of complex patients. I am going to bring up multiple aspects of patient care and I would like you to share whether you consider them to be a factor in your perception of a patient as complex.
5. Acuity of patient’s medical illnesses (for example a patient with heart failure with a very low ejection fraction or chronic obstructive lung disease on a high level of oxygen)
6. The number of chronic medical illnesses a patient carries.
7. The number of medications a patient is on for their different diagnoses.
8. The presence of mental illness in a patient with other chronic conditions.
9. Mental illness that is currently active (for example, a patient currently symptomatic with bipolar disorder or a patient who is actively suicidal)
10. The time it takes to care for the patient..
11. Patient financial issues such as money for transportation to doctor visits, co-pays, and/or for medications.
12. Patient issues with alcoholism or addiction
13. Social issues such as relationship stresses or other life stressors that may be affecting your patient.
14. Are than any other factors you feel I have left out that are important in the definition of complex patients?
15. Do you consider complex patients to be difficult to treat? Do you consider them the same as or different than “difficult patients”? If so, what are the differences?

(transitional sentence) The next couple of questions are about your preparation in caring for complex patients. We’re trying to understand what clinicians have been taught about caring for complex patients.

1. What training do you consider necessary to care for complex patients? Do you feel that you received this training? If so, from which settings?
2. --(probe) If difficulty recalling training, Was there a specific activity in medical school or residency where you learned to treat complex patients, i.e. clinic, inpatient rotations, rotations at the Veterans Administration, explicit didactic sessions on the care of patients with multiple chronic diseases, or specific training experiences after residency (conferences, didactics, peers at work?)?
3. How have you been trained to care for patients with mental illness? Again, was there a specific activity in medical school or residency where you learned to treat patients with mental illness, i.e. a psychiatric rotation in medical school, continuity clinic in residency, specific rotations in residency, didactics in medical school or residency—or specific training experiences after residency (conferences, didactics, peers at work?)?

**Patient Chart Exploration:**

Transition sentence: now I’d like to move on to some questions about the patient charts you chose to bring today.

Please refer to the patient charts you brought with you today. (physician participants should have 3 patient notes to reference for this section of interview. Please re-iterate that they are not to disclose any PHI. Explicitly let them know not to give the name, medical record number, date of birth, address or any other identifying information. )

1. What made you pick these three charts? (probes: how do these patients stand out for you? For some people, it is the 3 patients who they have the most difficulty (or dread) seeing; for others, it is patients they enjoy seeing because they are clinically stimulating.)
2. If they have a mental illness diagnosis, can you talk a bit about the role of mental illness/health in the care of these patients? (probes: what is it like for you to care for these patients? How do you think their mental illness might affect how you handle their care?)
3. Can you tell me about any other factors – such as age, gender, race, social issues – that you think might affect the care of these patients? (probes: Some physicians find that financial or insurance issues the care of these patients. Some also find that strong social support of family can greatly assist in their care.)
4. What do you find most enjoyable/rewarding in the care of these patients?
5. What do you find most frustrating / challenging in the care of these patients?
6. Is there anything you would like to add about the care of these patients? (probe: are there any experiences you have had with these patients we have not discussed that you feel are important? Is there a reason you chose these patient charts that we have not discussed?)

Thanks so much for bringing these charts and discussing these 3 patients with me. I just have 2 more questions for you, and then we are finished with the interview.

**Conclusive Questions:**

1. We also want to identify strategies for treating complex patients – especially those with mental illness- in primary care. Can you describe a recent situation in which someone (a colleague, or yourself) has handled a complex patient particularly well? What did they say and what did they do that makes you see it as being handled well? Can you tell me more about it?

I would like to thank you for your time. Your input is an invaluable resource. Someone from the study may contact you in the future for a short series of follow-up questions.
