# Supplementary material for: Designing the relational team development intervention to improve management of mental health in primary care using iterative stakeholder engagement
Source: BMC Fam Pract. 2019 Sep 6;20:124. doi: 10.1186/s12875-019-1010-z (PMC6728939; doi:10.1186/s12875-019-1010-z)
Supplement: Supplementary file 2 — Phase 1 Survey Instrument. (DOCx 115kb) [file 12875_2019_1010_MOESM2_ESM.docx]

**Guidelines:**

- You are not required to answer every question in the survey.
- There is no right or wrong answer.
- Please answer based on your current practice and understanding, unless otherwise indicated.
- All of your answers will be kept strictly confidential and will be reported only in summaries; i.e., with the responses of other participants.
- For each question, select one answer either by circling the number associated with your answer or marking the appropriate box.

1. **Please answer the following on a 0 – 10 scale regarding your confidence in the following domains of patient care. Circle 1 number for each question.**

| How confident are you that you can… | 0 (not at all confident) – 10 (extremely confident) | | | | | | | | | | |
| --- | --- | --- | --- | --- | --- | --- | --- | --- | --- | --- | --- |
| 1. diagnose hypertension? | 0 | 1 | 2 | 3 | 4 | 5 | 6 | 7 | 8 | 9 | 10 |
| 2. treat hypertension? | 0 | 1 | 2 | 3 | 4 | 5 | 6 | 7 | 8 | 9 | 10 |
| 3. diagnose chronic obstructive pulmonary disease? | 0 | 1 | 2 | 3 | 4 | 5 | 6 | 7 | 8 | 9 | 10 |
| 4. treat chronic obstructive pulmonary disease? | 0 | 1 | 2 | 3 | 4 | 5 | 6 | 7 | 8 | 9 | 10 |
| 5. treat two or more co-morbid chronic medical illnesses? | 0 | 1 | 2 | 3 | 4 | 5 | 6 | 7 | 8 | 9 | 10 |
| 6. diagnose major depression? | 0 | 1 | 2 | 3 | 4 | 5 | 6 | 7 | 8 | 9 | 10 |
| 7. treat major depression? | 0 | 1 | 2 | 3 | 4 | 5 | 6 | 7 | 8 | 9 | 10 |
| 8. diagnose generalized anxiety disorder? | 0 | 1 | 2 | 3 | 4 | 5 | 6 | 7 | 8 | 9 | 10 |
| 9. treat generalized anxiety disorder? | 0 | 1 | 2 | 3 | 4 | 5 | 6 | 7 | 8 | 9 | 10 |
| 10. diagnose bipolar disorder? | 0 | 1 | 2 | 3 | 4 | 5 | 6 | 7 | 8 | 9 | 10 |
| 11. treat bipolar disorder? | 0 | 1 | 2 | 3 | 4 | 5 | 6 | 7 | 8 | 9 | 10 |
| 12. manage an acutely suicidal patient? | 0 | 1 | 2 | 3 | 4 | 5 | 6 | 7 | 8 | 9 | 10 |
| 13. treat your patients who have both chronic medical and mental illness? | 0 | 1 | 2 | 3 | 4 | 5 | 6 | 7 | 8 | 9 | 10 |
| 14. have a productive conversation with a pulmonologist to care for a patient with chronic obstructive pulmonary disease? | 0 | 1 | 2 | 3 | 4 | 5 | 6 | 7 | 8 | 9 | 10 |
| 15. have a productive conversation with a psychologist to care for a patient with bipolar disorder? | 0 | 1 | 2 | 3 | 4 | 5 | 6 | 7 | 8 | 9 | 10 |
| 16. have a productive conversation with a psychiatrist to care for a patient with bipolar disorder? | 0 | 1 | 2 | 3 | 4 | 5 | 6 | 7 | 8 | 9 | 10 |
| 17. maintain ongoing trusting relationships with the patients in your panel? | 0 | 1 | 2 | 3 | 4 | 5 | 6 | 7 | 8 | 9 | 10 |
| 18. explain treatment options to your patient in a manner that ensures a high level of understanding by your patient? | 0 | 1 | 2 | 3 | 4 | 5 | 6 | 7 | 8 | 9 | 10 |
| 19. help your patient cope with her or his worries by explaining the current medical problem to her/him in a manner that facilitates coping? | 0 | 1 | 2 | 3 | 4 | 5 | 6 | 7 | 8 | 9 | 10 |
| 20. explain the possible benefits and risks to your patient of the recommended tests, procedures, and treatment options (including medications)? | 0 | 1 | 2 | 3 | 4 | 5 | 6 | 7 | 8 | 9 | 10 |
| 21. convey empathy to your patient regarding her/his problem? | 0 | 1 | 2 | 3 | 4 | 5 | 6 | 7 | 8 | 9 | 10 |

| How confident are you that you can… | 0 (not at all confident) – 10 (extremely confident) | | | | | | | | | | |
| --- | --- | --- | --- | --- | --- | --- | --- | --- | --- | --- | --- |
| 22. identify and pursue verbal cues given by your patient? | 0 | 1 | 2 | 3 | 4 | 5 | 6 | 7 | 8 | 9 | 10 |
| 23. identify and pursue non-verbal cues given by your patient? | 0 | 1 | 2 | 3 | 4 | 5 | 6 | 7 | 8 | 9 | 10 |
| 24. communicate effectively with your patient even though you find her/him to be rather difficult? | 0 | 1 | 2 | 3 | 4 | 5 | 6 | 7 | 8 | 9 | 10 |
| 25. actively involve your patient in the process of making treatment-related decisions? | 0 | 1 | 2 | 3 | 4 | 5 | 6 | 7 | 8 | 9 | 10 |
| 26. secure your patient’s commitment to try to follow the treatment plan that you developed with your patient? | 0 | 1 | 2 | 3 | 4 | 5 | 6 | 7 | 8 | 9 | 10 |
| 27. use the last few minutes of the encounter to summarize the important issues discussed during the encounter? | 0 | 1 | 2 | 3 | 4 | 5 | 6 | 7 | 8 | 9 | 10 |
| 2) For the next 7 questions, refer to the following clinical scenario:  Please answer these questions even in you do not work in a team setting. Imagine you work in a primary clinic with a team-based approach to care. There are 3 clinical teams in the clinic. Your team includes: 1 nurse, 1 care manager, 5 medical assistants, 1 nurse practitioner, and 4 physicians.  The *care manager* is a nurse whose primary responsibility is to use databases to track the clinical care of patients in your team with chronic medical or mental illnesses.  The *care manager* focuses on the following chronic conditions: depression, diabetes mellitus, and hypertension. The *care manager* uses evidence-based treatment protocols to make treatment adjustments for these conditions. The *care manager* consults with you, the primary care provider, on patients who do not respond to treatment as expected.  Working as a physician in this clinical setting, how confident are you that you would be able to… | | | | | | | | | | | |
|  | **0 (not at all confident) – 10 (extremely confident)** | | | | | | | | | | |
| 1. rely on the care manager to work with your patients to develop specific plans to meet their goals for their chronic medical or mental illness(es)? | 0 | 1 | 2 | 3 | 4 | 5 | 6 | 7 | 8 | 9 | 10 |
| 2. rely on the care manager to contact your patients to monitor their progress toward meeting their goals for their chronic medical or mental illness(es)? | 0 | 1 | 2 | 3 | 4 | 5 | 6 | 7 | 8 | 9 | 10 |
| 3. rely on the care manager to contact patients in your panel with diabetes who are overdue for their HbA1c to schedule them to come in to clinic for follow-up testing? | 0 | 1 | 2 | 3 | 4 | 5 | 6 | 7 | 8 | 9 | 10 |
| 4. collaborate with the care manager who uses treatment algorithms to make medication adjustments to improve blood pressure control in your patients with uncontrolled hypertension? | 0 | 1 | 2 | 3 | 4 | 5 | 6 | 7 | 8 | 9 | 10 |
| 5. collaborate with the care manager who uses treatment algorithms to make medication adjustments to improve symptom control in your patients with uncontrolled major depression? | 0 | 1 | 2 | 3 | 4 | 5 | 6 | 7 | 8 | 9 | 10 |
| 6. encourage team members to contribute to creative solutions to improve patient care within your practice? | 0 | 1 | 2 | 3 | 4 | 5 | 6 | 7 | 8 | 9 | 10 |
| 7. establish a positive atmosphere among the members of the clinical team? | 0 | 1 | 2 | 3 | 4 | 5 | 6 | 7 | 8 | 9 | 10  **5** |

**7) The following questions ask your opinion about working with a team of providers to care for your patient. Please indicate your level of agreement with each of the following statements**

**by circling the number that best reflects your level of agreement for each**

**statement.**

**8) The following questions ask about various strategies that you might use in patient care. Each strategy is answered on a 5-point scale, which represents how fully you implement each strategy in your current practice setting.**

**Circle the number that best reflects the completeness of implementation.**

**If a strategy is completely implemented, it means it is now routine practice.**

**9) Please indicate your level of agreement with each of the following statements. Circle 1 option. Please answer the following questions in the context of your primary clinical practice site. The following questions ask about your “team”.**

**For these questions, please think of your “team” as the health care providers, nurses, and other staff at your practice or clinic.**

| **Statement** | **Strongly Disagree** | **Disagree** | **Neither Agree nor Disagree** | **Agree** | **Strongly Agree** |
| --- | --- | --- | --- | --- | --- |
| We have a "we are in it together" attitude. | 1 | 2 | 3 | 4 | 5 |
| People keep each other informed about work related issues in the team. | 1 | 2 | 3 | 4 | 5 |
| People feel understood and accepted by each other. | 1 | 2 | 3 | 4 | 5 |
| There are real attempts to share information throughout the team. | 1 | 2 | 3 | 4 | 5 |
| There is a lot of give and take. | 1 | 2 | 3 | 4 | 5 |
| We keep in touch with each other as a team. | 1 | 2 | 3 | 4 | 5 |

**10) Which of these resources do you currently have in your clinical practice site?**

| **Resource** | **Yes** | **No** |
| --- | --- | --- |
| Social worker | ❑ | ❑ |
| Case manager | ❑ | ❑ |
| Health psychologist | ❑ | ❑ |
| Pharmacist | ❑ | ❑ |
| Electronic Medical Record | ❑ | ❑ |
| Patient Registries | ❑ | ❑ |
| Access to psychiatry consultation | ❑ | ❑ |
| Access to psychology consultation | ❑ | ❑ |

**11) Which best describes your primary clinical practice site? (Check 1 option)**

| University-based practice | ❑ |
| --- | --- |
| Non-academic hospital-based practice | ❑ |
| Managed Care Organization | ❑ |
| Private practice | ❑ |
| Community health center | ❑ |
| Veterans Administration or Military Clinic | ❑ |
| Other | ❑ |

**12) In what type of setting is your practice located? (Check 1 option)**

| Urban, inner city | ❑ |
| --- | --- |
| Urban, non-inner city / suburban | ❑ |
| Rural | ❑ |

**13) Including yourself, how many providers (including nurse practitioners and physician assistants but not including nurses) work in your practice?**

**If you work in an institution with many primary care clinics, include only your clinic site.**

**(Check 1 option)**

| 1 (just myself) | ❑ |
| --- | --- |
| 2 - 5 | ❑ |
| 6 - 10 | ❑ |
| ≥ 11 | ❑ |

**14) What is your primary professional role?**

**(Check 1 option)**

| Clinician | ❑ |
| --- | --- |
| Clinician - Educator | ❑ |
| Clinician - Researcher | ❑ |
| Clinician - Administrator | ❑ |

**15) What is your medical specialty?**

**(Check 1 option)**

| Family Medicine | ❑ |
| --- | --- |
| Internal Medicine | ❑ |

**16) How many years has it been since you completed residency?**

**(Check 1 option)**

| < 5 | ❑ |
| --- | --- |
| 5 - 9 | ❑ |
| 10 - 19 | ❑ |
| ≥ 20 | ❑ |

**17) What is your gender?**

**(Check 1 option)**

| Male | ❑ |
| --- | --- |
| Female | ❑ |

**18) What is your race?**

**(Check 1 option)**

| African-American / Black | ❑ |
| --- | --- |
| Caucasian / White | ❑ |
| Asian (includes Southeast Asian, Indian) | ❑ |
| Pacific Islander / Native Hawaiian | ❑ |
| American Indian / Alaskan Native | ❑ |
| Multiple Races | ❑ |
| Other | ❑ |

**19) What is your ethnicity?**

**(Check 1 option)**

| Hispanic | ❑ |
| --- | --- |
| Non-Hispanic | ❑ |

Please place your completed survey in the accompanying stamped mailing envelope, seal it, and place it in the mail.

THANK YOU!

Q1: Chronic Medical Illness Self-Efficacy Scale (questions 1-5, 14)

Mental Illness Management Self-Efficacy Scale (questions 6-13,15-16) and modified Communication Skills Self-Assessment (questions 17-27)[^1^](#_ENREF_1)

Q2: Team-based Care Self-efficacy Scale

Q3: Treatment knowledge (modified)[^2^](#_ENREF_2)

Q4-Q6: Clinical experience (modified)[^2^](#_ENREF_2)

Q7: Attitudes toward Health Teams Scale[^3^](#_ENREF_3)

Q8: Team Approach Subscale of the Practice Monitor(modified)[^4^](#_ENREF_4)

Q9: Team Climate Inventory (modified)[^5^](#_ENREF_5)^,^[^6^](#_ENREF_6)

Q10-Q19: Resources, clinical practice, and demographic questions

**1.** Ashbury FD, Iverson DC, Kralj B. Physician Communication Skills: Results of a Survey of General/Family Practitioners in Newfoundland. *Med Educ Online.* 2001;6(1).

**2.** Katerndahl D, Ferrer RL. Knowledge About Recommended Treatment and Management of Major Depressive Disorder, Panic Disorder, and Generalized Anxiety Disorder Among Family Physicians. *Prim Care Companion J Clin Psychiatry.* 2004;6(4):147-151.

**3.** Heinemann GD, Schmitt MH, Farrell MP, Brallier SA. Development of an Attitudes Toward Health Care Teams Scale. *Evaluation & the health professions.* Mar 1999;22(1):123-142.

**4.** Comprehensive Primary Care Practice Monitor. <http://cufamilymedicine.org/evaluation_hub/resources-2/comprehensive-primary-care-practice-monitor/>. Accessed March 4, 2016.

**5.** Anderson NR, West MA. Measuring climate for work group innovation: development and validation of the team climate inventory. *Journal of Organizational Behavior.* 1998;19(3):235-258.

**6.** Kivimaki M, Elovainio M. A short version of the Team Climate Inventory: Development and psychometric properties. *Journal of Occupational and Organizational Psychology.* 1999;72(2):241-246.
