# Supplementary material for: Designing the relational team development intervention to improve management of mental health in primary care using iterative stakeholder engagement
Source: BMC Fam Pract. 2019 Sep 6;20:124. doi: 10.1186/s12875-019-1010-z (PMC6728939; doi:10.1186/s12875-019-1010-z)
Supplement: Supplementary file 3 — Phase 2 Interview Guide. (DOC 39kb) [file 12875_2019_1010_MOESM3_ESM.doc]

*Interview Guide*

**Project Title:** Primary Care Physician Care of Complex Patients with Mental Illness

**Principal Investigator:** Danielle Loeb, MD

**COMRIB Protocol:** 14-0359

**Version Date:** 6/15/2015

In this study we are seeking your opinions about a planned intervention at a similar clinic.

We are speaking with you as the local experts on how the intervention will work in a clinic like yours. We are seeking your honest opinions. There are no right or wrong answers.

*Before we start, I need to make sure you are aware of the following:*

- *Your participation is voluntary, you do not have to answer any question that makes you uncomfortable, and you may stop at anytime.*
- *Your responses will be recorded in a manner that will maintain your anonymity and confidentiality.*
- *If you have any concerns about this study, you may contact the Primary Investigator –Danielle Loeb or COMIRB (*303-724-0155).

*[Obtain verbal consent] Do you consent to go ahead with the interview?*

*[If no] Thank you for your time.*

*[If yes] Do you have any questions before we begin?*

***INTRODUCTION:*** *We will pass out the draft pilot Tailored Practice Facilitation (TPF) intervention (Attachment A).* Please take a moment to look over the draft pilot intervention. We will begin the focus group in a few minutes. We expect this discussion to take about 45 minutes.

***BEGIN RECORDING***

**Opening Questions (approx 10 min):**

1. Give a general summary of the intervention.
2. Broadly speaking, what do you think about the Tailored Practice Facilitation (TPF) intervention?
3. ---(probe) If people do not start discussing the TPF intervention—ask/explore if the they would find it useful to spend one-on-one time during their clinic day with a health psychologist with training in practice change.

**Specific Aspects of TPF Intervention (approx 30 min):**

1. I would like to explore the planned intervention in a little bit more detail: First, I would like to explore the planned didactics.
   1. Team Care Didactics: elicit general feedback

--prompts:

- - - - 1. -Do you think these are the most important concepts with respect to team-based care?
        2. What concepts would you add or take away?
        3. Do you have any suggestions for teaching team care concepts in a way that would be meaningful for practicing PCPs?
  1. Mental Health Didactics: elicit general feedback

--prompts:

- - - - 1. -Do you think these are the most important topics in mental health for PCPs?
        2. What topics would you add or take away?
        3. Do you have any suggestions for mental health topics in a way that would be meaningful for practicing PCPs?

1. Now I would like to hear your feedback on the experiential intervention with the health psychologist. We plan to have the health psychologist spend 2 clinic sessions with PCP participants and join them in their visits with their complex patients (those with a mood disorder and a chronic medical illness). They will also attend team meetings with PCP participants. They will give suggestions on ways for PCPs to engage the team in the care of patients; communication strategies with patients and with the team; help PCPs problem-solve regarding improvements within their team to help with the care of their complex patients.
   1. Elicit general feedback on intervention

--prompts

1. Would you consider this intervention worth your time?
2. Would you use the time of a health psychologist differently (other than patient care)?
3. Would you think it would be useful to have the health psychologist to train a team-member to continue their coaching activities?

**Conclusive Questions (approx 5 min):**

1. Do you have any other suggestions for the intervention?
2. If you think of further suggestions, please do not hesitate to contact either Sue Felton or I (give out contact information).
3. If you are interested helping in future planning with this intervention please let me know.

I would like to thank you for your time. Your input is an invaluable resource.
